# Emotional imagination of negative situations: Functional neuroimaging in anorexia and bulimia

**DOI:** 10.1371/journal.pone.0231684

**Published:** 2021-04-09

**Authors:** Federico D’Agata, Paola Caroppo, Angela Spalatro, Luca Lavagnino, Giovanni Abbate Daga, Andrea Boghi, Mauro Bergui, Alessandro Cicerale, Benedetto Vitiello, Secondo Fassino, Birgit Derntl, Federico Amianto

**Affiliations:** 1 Department of Neurosciences, University of Torino, Turin, Italy; 2 Computational Imaging Group for MR diagnostics & therapy, Center for Image Sciences, University Medical Center Utrecht, Utrecht, The Netherlands; 3 Fondazione IRCCS Istituto Neurologico Carlo Besta, Milan, Italy; 4 UT Center of Excellence on Mood Disorders, Department of Psychiatry and Behavioral Sciences, UT Houston Medical School, Houston, Texas, United States of America; 5 ASL TO2 San Giovanni Bosco Hospital, Turin, Italy; 6 Department of Public Health and Pediatric Sciences, University of Torino, Turin, Italy; 7 Department for Psychiatry and Psychotherapy, University Hospital Tübingen, Tübingen, Germany; National Institutes of Health, UNITED STATES

## Abstract

**Aim:**

The present study aims to extend the knowledge of the neural correlates of emotion processing in first episode subjects affected by anorexia nervosa (AN) or bulimia nervosa (BN). We applied an emotional distress paradigm targeting negative emotions thought to be relevant for interpersonal difficulties and therapeutic resistance mechanisms.

**Methods:**

The current study applied to 44 female participants with newly diagnosed AN or BN and 20 matched controls a neuroimaging paradigm eliciting affective responses. The measurements also included an extensive assessment comprising clinical scales, neuropsychological tests, measures of emotion processing and empathy.

**Results:**

AN and BN did not differ from controls in terms of emotional response, emotion matching, self-reported empathy and cognitive performance. However, eating disorder and psychopathological clinical scores, as well as alexithymia levels, were increased in AN and BN. On a neural level, no significant group differences emerged, even when focusing on a region of interest selected a priori: the amygdala. Some interesting findings put in relation the hippocampal activity with the level of Body Dissatisfaction of the participants, the relative importance of the key nodes for the common network in the decoding of different emotions (BN = right amygdala, AN = anterior cingulate area), and the qualitative profile of the deactivations.

**Conclusions:**

Our data do not support the hypothesis that participants with AN or BN display reduced emotional responsiveness. However, peculiar characteristics in emotion processing could be associated to the three different groups. Therefore, relational difficulties in eating disorders, as well as therapeutic resistance, could be not secondary to a simple difficulty in feeling and identifying basic negative emotions in AN and BN participants.

## Introduction

Anorexia nervosa (AN) and Bulimia nervosa (BN) are the two major Eating Disorders (ED): serious and complex psychiatric conditions with a multifactorial biopsychosocial pathogenesis, often characterized by a chronic and disabling course and only partial therapeutic success [1,2]. Young girls are especially affected by AN, which is the pathology with the highest mortality risk and the lowest response to treatment across ED [3]. Prioritizing the treatment of symptoms results in better outcomes in BN and allows dealing with the main cause of mortality in AN [4]. It remains controversial whether doing so ignores core psychopathological elements, linked to more complex symptoms and long-term outcomes such as relationship difficulties or impairments in affect regulation, reflective functioning, and coherence of mind [5]. Psychotherapeutic treatment often focuses on these aspects and therapists are frequently faced with marked difficulty in engaging subjects affected by AN and maintaining treatment adherence [6]. In BN the difficulties are related to coping with high emotional arousal when facing social and affective stimuli. These difficulties also challenge a complex therapeutic approach [7].

The problems of individuals affected by ED in social interactions and psychotherapeutic engagement are indicators of serious difficulties in the management of interpersonal relationships, as well as emotional dysregulation [8]. Although not included in the current diagnostic criteria of ED (DSM-5 and ICD-10), emerging evidence points to deficits in socioemotional functioning [9]. Consequently, several modern therapeutic models incorporate the role of emotional difficulties, social anxiety and poor social support in the maintenance of the disorder [8].

Empathy represents a core function for social coherence and building relationships [10]. Based on the abovementioned socio-emotional difficulties and related problems in ED, one may assume that empathy and basic emotion processing are systematically altered in ED and their impairments potentially represent relevant risk factors. Several studies applied self-reported empathy measures or assessed emotion recognition performance but reported mixed results [11–14]. Thus, it is unclear how much emotion processing, empathy and social competencies are affected in ED and what mechanisms mediate the insurgence of relational difficulties.

Most neuroimaging studies in the field of ED have investigated the neurobiological correlates of body shape, reward and food stimuli [15,16], while the number of studies focusing on emotion/empathy is still scarce and almost limited to the functional magnetic imaging (fMRI) correlates of implicit and/or explicit face emotion processing in patients affected by AN [17–22]. A greater response in AN to facial emotional expressions was observed, especially if attending to own or infant face images [18,20,22], but these findings were not always confirmed [21]. Amygdala activation during stimuli observation was reported as enhanced [17,20] or reduced [19], but the exact mechanisms are complex and the experimental setups difficult to compare. Generally enhanced prefrontal activity was observed and interpreted as a down-regulation mechanism to suppress elevated limbic reactivity [17,20].

In BN much less is known regarding the neural circuits underlying emotion processing. Reduced activation of the amygdala has been reported and interpreted as deficits in emotional appraisal and processing in BN patients [16].

Only a small number of neuroimaging study in the field adopted a transdiagnostic approach by including both AN and BN [23–25]. Such an approach can test the hypothesis of a common or different neural substrate in ED. As the neurobiology of ED is still under study and very complex, it is unclear if the deficits are due to abnormal feeding behavior or if they are already present before pathological behaviors emerge. Modern pathogenic models postulate that social and familial factors, together with an altered brain functional maturity [26,27], have a role in the genesis and maintenance of the disturbances. Alterations in the microbiome, in the neurotransmitters, in the white and gray matter volumetry, in the functional and anatomical connectivity between cerebral areas have been reported. These alterations mostly seem to regress with the normalization of feeding behavior, and they seem to be related to a common substrate linked to reward networks (the striatum). On the other side, emotional processing in ED has been usually investigated from the clinical or therapeutic side, but it was scarcely studied from a neurobiological point of view.

Therefore, the present work aims to expand this knowledge using behavioral measures and an fMRI paradigm able to tap into emotional processes judged to be important for empathy. In particular, we used one of the 3 tasks developed from the tripartite empathy model of Decety [10]. The tasks are facial emotion recognition, affective responsiveness and perspective-taking. We discarded the first, as it has already been extensively used by other papers investigating ED and choose the second task as it is more focused on emotion processing and therefore relevant for our research question. This task has been adopted in studies investigating psychiatric (but non-ED) groups, and it asked participants to consider themselves in a negative emotional situation and then choose which facial emotional expression they would show.

The studies investigating the task we used in other groups reported consistent amygdala activations [28–30]. Furthermore, it is well known that the amygdala is a key area involved in emotion processing, as reported also by many fMRI studies [19,31–33].

The amygdalae are part of the limbic areas and contain approximately 12 different regions, each of which can be further divided into several subregions [31,34]. This complex structure has been implicated in a wide variety of brain activities such as emotional regulation and other cognitive functions. The most current view emphasizes its involvement in negative emotions, especially fear, and connects them with other cognitive processes, such as memory and associative learning [31,32,35]. In particular, the amygdala has been implicated to have a role in empathy and empathy for others’ pain [36–38].

For these reasons, we decided to select the amygdala as our a priori ROI of interest for this study, in addition to the usual whole-brain analyses.

The present study aims to extend the knowledge of the neural correlates of emotion imagination and matching in first-episode young women affected by AN or BN, using an fMRI paradigm focusing on response to negative emotions, as these could be relevant for interpersonal problems and therapeutic resistance mechanisms [8]. The importance of selecting first-episode participants derives from the aim of excluding secondary and/or chronic effects of the disorders and the effects of prolonged or repeated therapeutic interventions.

We compared the performance of women affected by AN or BN and a group of matched healthy controls with an extensive assessment that included clinical scales, neuropsychological tests, and self-report questionnaires of emotion processing and empathy and with fMRI. Based on previous studies we hypothesized: i) behavioral and/or self-reported differences in emotion processing/empathy measures between the three groups, ii) group differences for the activation of the amygdala, and iii) a critical contribution of neuroimaging data to distinguish between the three groups as compared to the behavioral or self-report measures, as it should be a less biased and more sensitive measurement tool.

## Materials and methods

### Sample

**Fig 1:**
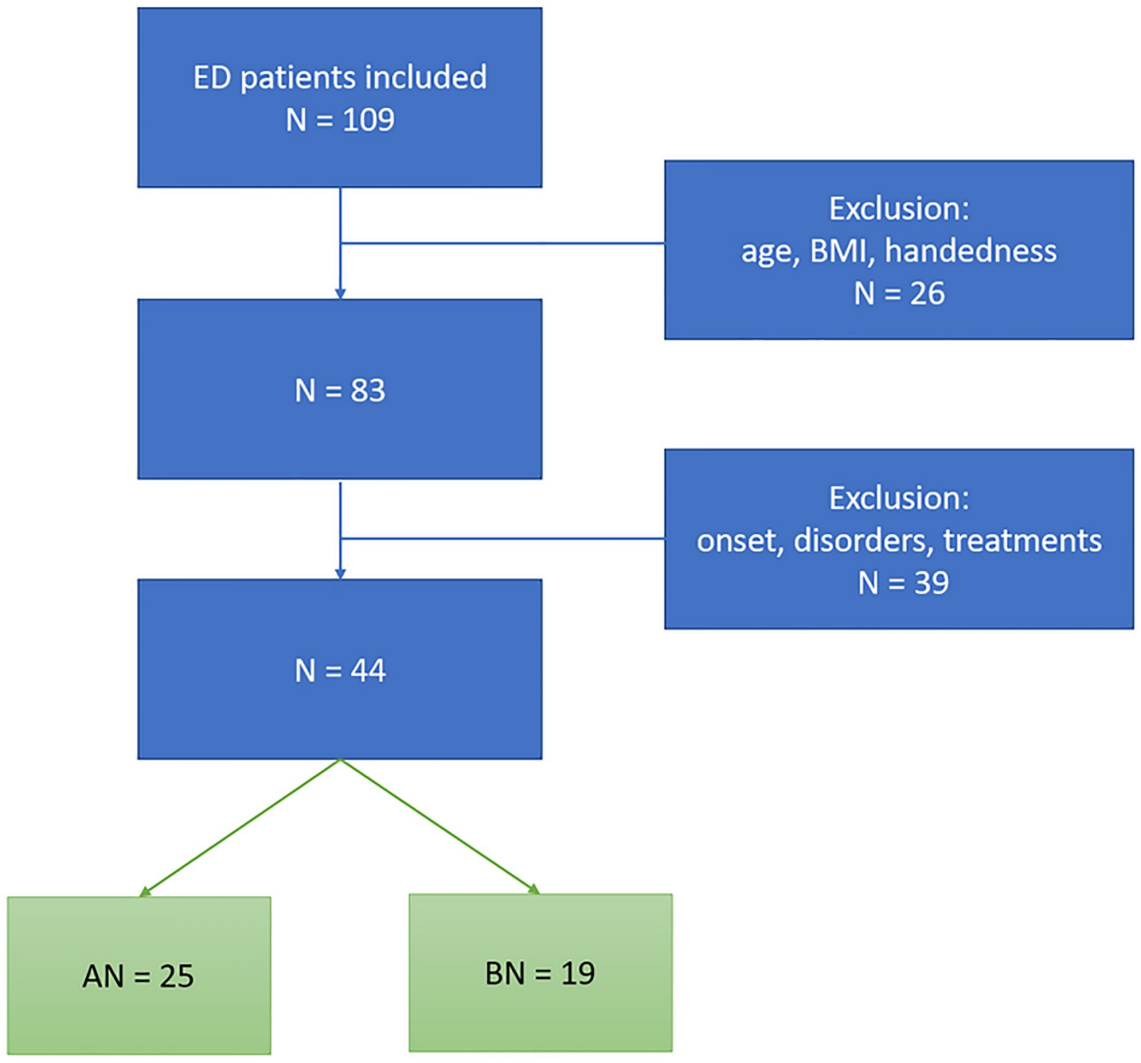
Recruitment flowchart.

Twenty-five female individuals affected by AN (20 restricting, 5 binge/purging) and 19 by BN (14 purging, 5 not purging), were enrolled from the outpatient service of the Pilot Centre for the Diagnosis and Treatment of Eating Disorders of the Department of Neuroscience, “AOU Città della Salute e della Scienza” of Turin, Italy. ED was diagnosed using the Structured Clinical Interview for DSM-IV-TR (SCID). Each patient was assessed by a psychiatrist contributing to this paper (LL, AS or FA). The inclusion criteria for the study were: female sex; age 16–30 years; right-handedness (assessed by Edinburgh Handedness Inventory); body mass index (BMI) from 15.0 to 17.5 for AN and from 19.0 to 25.0 for BN; no past or present mental disorder except for the current ED first-episode; no axis II disorders (assessed by SCID II, [39]; no current or past pharmacological treatment; no drug or alcohol abuse; no history of diabetes or other somatic diseases, no past or present psychotherapy treatment and duration of symptoms shorter than 2 years. From a global sample of 109 assessed ED participants, only 44 met the inclusion criteria and were finally enrolled in the present study (see Fig 1 for the recruitment flowchart).

Twenty healthy women were recruited as controls (CN). The inclusion criteria for the CN group were having a normal BMI (between 18.5 and 25), the exclusion criteria were having a current or lifetime mental disorder. The absence of neuropsychiatric and cognitive problems in CN was assessed by SCID interviews and neuropsychological testing. Possible control subjects were recruited through local advertisement, and for each AN or BN patients a matched control was drawn from the list. Age was the primary criterion for the matching, and efforts were taken to match education level.

All participants gave their written informed consent to the study. For minors (3 AN, 1 BN), written informed consent was obtained from parents. The study was approved by the local Ethics Committee [Comitato Etico Interaziendale A.O.U. Città della Salute e della Scienza di Torino—A.O. Ordine Mauriziano—A.S.L. Città di Torino, approval #12042010].

### Clinical and self-report data

The clinical assessment included the *Eating Disorder Inventory* 2 (EDI-2) [40], the *Symptom Checklist-90* (SCL-90) [41], the *Empathy Quotient* [42] and the *Toronto Alexithymia Scale* (TAS-20) [43] Detailed information on the scales can be found in the supplementary material (section S1.1) in S1 File. Three AN did not turn in EDI-2, Empathy Quotient, and SCL-90 scales. Two AN did not return the completed TAS-20 forms.

### Neuropsychological tests

All participants performed a comprehensive neuropsychological testing battery assessing attention, memory and executive functions to check the assumption that the groups did not differ significantly in cognitive performances, therefore helping us discard alternative explanations for our neuroimaging results. Tests were administered by a trained neuropsychologist, helped by collaborators. Detailed information can be found in the supplementary material (section S1.1) in S1 File. One BN and one CN subject did not carry out the Digit tests, two CNs did not carry out the Wisconsin Card Sorting Test, and one CN did not carry out the Corsi span.

### MRI acquisition

MRI data were collected on a Philips Achieva 1.5T scanner. First, participants underwent the affective responsiveness task that comprised 480 continuous gradient-recalled EPI volumes (TR = 2300ms, TE = 40ms, FA = 90°, 30 axial slices, matrix = 128x128, slice thickness = 4mm, no gap, voxel size = 1.8x1.8x 4mm^3^, field of view = 23cm). After fMRI, anatomical high-resolution images were acquired using T1-weighted 3D Turbo Field-Echo sequence (matrix = 256x256, 190 contiguous sagittal slices, TR = 7ms, TE = 3ms, TFE shots = 89, voxel size 1x1x1mm^3^).

#### Affective responsiveness fMRI paradigm

We used a modified and shortened version of a previously tested fMRI paradigm [28–30,44]. Forty short written sentences were presented, describing real-life situations inducing anger, fear, disgust (e.g., ‘You are walking in a meadow and step on dog excrement’ for disgust) or containing a neutral content (e.g., ‘You are on the couch watching television’). As reported previously, stimuli were validated by independent female and male raters [29] and only stimuli that were classified as belonging to one emotional category (>70%) were selected for the study. We presented 10 sentences per condition (disgust, anger, fear, neutral). Participants had to imagine how they would feel if they were experiencing those situations. We were particularly interested in the emotional response to negative situations. Stimuli were presented for 7 sec. After emotion induction participants were presented with two facial expressions of other people (actors), one with the same emotion as the induction (correctly matched answer) and the other displaying a wrong emotion, chosen randomly from the other options. Participants were asked to choose between the two and they had a maximum of 7 seconds to respond.

The instructions given to the participants were the translation in Italian of the ones found in [41]: “Come si sentirebbe in questa situazione? Quale espressione emotiva mostrebbe?” (How would you feel in this situation? Which emotional expression would you show?”.

The response was followed by 7 sec of inter-trial-interval (cross fixation), then another trial started. The number of correct answers for the matching of emotional sentences and faces was recorded as a proxy of emotion processing (score 0–30). A right answer likely means a correct emotional response to the imaginary situation, correct identification of the emotions in the two displayed faces, correct comparison and matching. After a neutral situation, participants were presented with two neutral faces, one male and one female, and they had to identify the female within 7 seconds. The number of correct answers was recorded as a proxy of the sustained attention and active participation to the task (score 0–10).

We did not use an explicit jitter, but the variable time at which the participants answered (they were free to press the button when they wanted) naturally created time variability between the different parts of the fMRI task (imagination and emotion matching).

**Fig 2:**
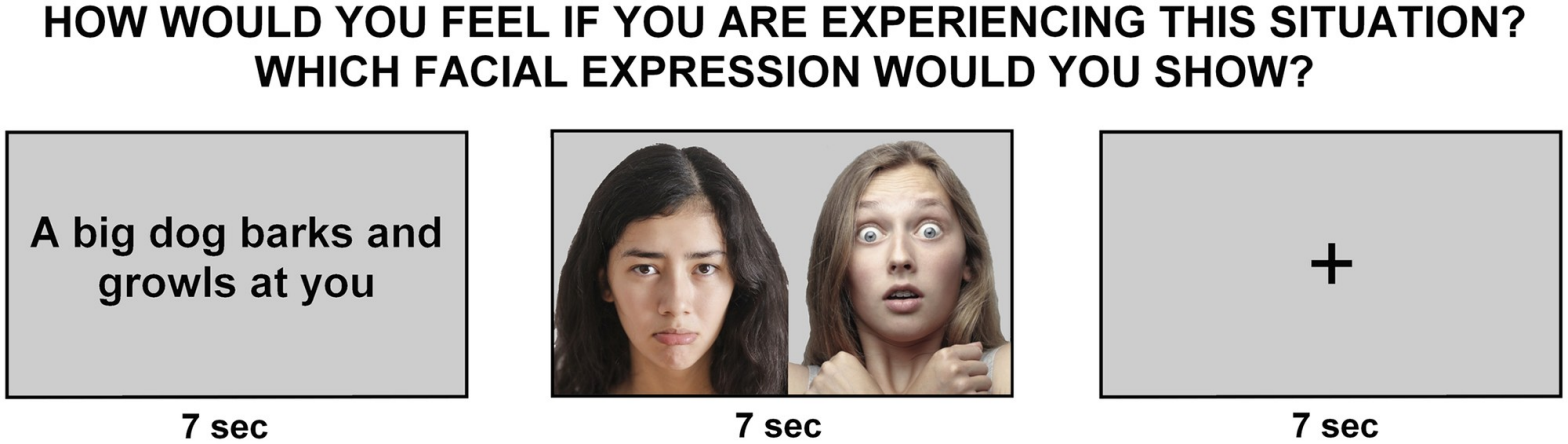
Illustration of the emotional response task. In every trial a short sentence with negative or neutral valence was presented, after which the participant was asked to choose between two emotional face stimuli of other people (one correctly matched and one wrongly) and had to select the facial expression that best matched the induced feelings. The paradigm consisted of 30 negative emotion trials (10 anger, 10 fear, 10 disgust) and 10 neutral trials. The stimulus displayed here is not one of the originals used in the experiment, but a high-resolution reproduction suitable for printing and display. Image attribution: Andrea Piacquadio/Pexels (left and right faces).

The order presentation of neutral and emotional stimuli was counterbalanced across participants. Fig 2 illustrates an example of the task. Further information on the experimental design can be found in the supplementary material (section S1.2) in S1 File.

#### Statistical comparison of behavioral and questionnaire data

Statistical analyses were performed using IBM SPSS Statistics for Windows (Version 20.0. Armonk, NY: IBM Corp.) and level of significance was set to p < .05 corrected with False Discovery Rate (FDR q = .05) to account for multiple comparisons [45]. Behavioral and questionnaire data were compared using one-way ANOVAs with GROUP as between-subjects factor. When needed, we performed a repeated measure ANOVA (rmANOVA) with emotion as within-subjects factor (see the results in the Behavioral performance during affective responsiveness task section). We used Tukey post-hoc tests to disentangle group differences when the main effect was significant. Effect sizes are reported using partial eta squared (η^2^).

#### FMRI data processing

Functional data were preprocessed using SPM8 (http://www.fil.ion.ucl.ac.uk/spm) on MATLAB 7.5 environment. Images were motion corrected, normalized into the standardized stereotaxic MNI space using T1 coregistered images and spatially smoothed using an isotropic Gaussian kernel with a full-width-at-half-maximum (FWHM) of 8mm.

The estimation of head motion was tested with a procedure previously described by Yuan et al. [46]. The magnitude of head motion for six parameters (three for shift and three for rotation) was obtained for each participant and the averaged head motion parameters were calculated. An ANOVA was computed to test for differences between the groups. The result was non-significant, both for rotation (p = .8; average movement × TR = 0.05°) and shift (p = .6; average movement × TR = 0.1mm). Yuan and colleagues suggest the exclusion of participants with head motion exceeding 4 SDs (in our sample: rotation>0.1°; shift>0.2mm). None of our participants exceeded this threshold; therefore, data of all women were included in the analyses.

*Task-based fMRI analysis*. For this event-related design, each stimulus type was modeled with a separate regressor convolved with the canonical hemodynamic response function (HRF). Also, responses to the two different tasks were modeled as separate regressors. The participants gave correct answers in more than 85% of the trials, and the number of wrong responses did not differ significantly between groups (p = .5). The time-series movement estimated parameters (3 translations, 3 rotations) were included as covariates of no interest and the HRF time derivative was included in the model to address slice-timing correction.

Statistical analysis was performed with a two-step hierarchical estimation. At a first individual level, we saved three contrast images for every participant: disgust (D), anger (A), fear (F) compared to the neutral condition N (D>N, A>N, F>N, the contrasts compared 10 emotional trials with 10 neutral trials for each of the three conditions). We included the response-related regressors (button press) only in first level models, but not in the second level models, as we focused on the neural activation during imagined situations. Contrast images from all participants were included in three second-level random-effects analysis to detect group differences, never mixing D, A and F or overrepresenting in the same model the N trials. The models were three one-way ANCOVAs with GROUP as a between-subjects factor (AN, BN, CN) and controlling for atrophy. As cerebral atrophy could be a confounding factor in ED, as previously reported [47], we used VBM8 toolbox [48] to automatically extract gray matter (GM), white matter (WM) and cerebro-spinal fluid (CSF) volumes of all the participants. We compared parameters between groups including the significantly different brain volumes (CSF) as a covariate of no interest. Statistical inferences were performed by applying the Random Field Theory. Maps were thresholded at the p < .05 FWE cluster-level corrected (FWEc, meaning uncorrected p < .001, filtered for small clusters or cluster extent>FWE cluster size threshold).

*Multi-voxel pattern analysis*. We performed a multi-voxel pattern analysis (MVPA), in which we used the 40 first-level beta maps derived from the task-based fMRI analysis (one for each trial) to predict the presented stimulus emotion’s category. The conditions used were: D, F, A, N, each repeated 10 times; the classification performance was tested vs. 25% random accuracy. This was done to adopt a more powerful multivariate technique able to detect differences between groups in emotion imagination. We used The Decoding Toolbox v 3.999 [49], performing a whole-brain analysis using all the data sets with leave one out as the cross-validation method. The correct labels were the emotions induced by the sentences during the affective responsiveness task. The outputs of the analysis were the Support Vector Machine (SVM) weights and the predicted labels. The SVM weights are related to the model classification capacity of a feature, in our case, of a voxel. To identify the most important decoding areas for the 3 groups, we then performed a second-level analysis on the SVM weights, running an ANOVA with GROUP as between-subjects factor, and we analyzed the areas significant for the three groups and the pairwise differences between them adopting the same statistical thresholds as in the task-based fMRI analysis (p < .05 FWE cluster-level corrected). Results must be taken with care because although all the voxels contribute to the decoding of the classes, in some cases, only a subsample of them contains neural activations related to the task [49].

*Confusion matrix*. For each group, we used the output of the MVPA to create a mean confusion matrix (predicted vs. real emotions displayed in the stimulus) and we performed all the pairwise comparisons for every emotion using multiple binomials test [50]. We applied the Sidak correction to the obtained p values to control for multiple comparison errors (*n* = emotions x comparisons = 4 x 6).

*Emotion ROI analysis*. We performed ROI analyses separately on centromedial (CM) and basolateral (BL) left and right amygdala. These regions were selected a priori as they have been consistently reported as key regions in emotion processing with important specific properties, as CM (in particular the central portion) is considered to be the main output area and BL the principal input area of the amygdala during aversive stimuli presentation.

We also selected ROIs obtained from the group differences that emerged from the MVPA analysis. To do so, we used the MarsBaR region of interest toolbox for SPM (http://marsbar.sourceforge.net). The ROIs were delineated using the Melbourne Subcortex Atlas [51] in the MNI space, and ROIs mean signals were extracted. To perform the analysis, we used the same ANCOVA model adopted for the whole-brain analysis previously reported for every ROI separately. We applied the Sidak correction [52] to control for multiple comparison errors *(n* = number of tests, corrected threshold = $\alpha_{SID}=1-{\left(1-0.05\right)}^{\frac{1}{n}}$).

*Relationship between fMRI and clinical data*. We correlated the beta maps of the subjects computed from the first level analysis in the task-based fMRI analysis with anxiety and depression scores obtained from the SCL-90, with the subscales of the EDI-2, with the TAS-20, with the Empathic Quotient, and with the fMRI affective responsiveness scores, adopting the same statistical thresholds as in the task-based fMRI analysis (p < .05 FWE cluster-level corrected) and using the CSF volume as covariate, to control for atrophy. The model included the group factor, the mentioned scores along with the interaction effect between the group factor and the scores.

## Results

### Subjects data

#### Demographic, self-report and brain volume data

The demographic and clinical data are shown in Table 1. Groups did not differ in terms of age as well as educational level. However, as expected given the nature of the disease, the BMI of AN patients was significantly lower than BN and CN, while BN and CN did not differ from each other. The duration of the disease was similar for AN and BN. Regarding brain atrophy, the CSF global volume was increased in AN compared to both BN (p < .010) and CN (p < .002), while BN and CN did not differ (p < .895).

**Table 1:** Demographic and clinical data separately for the three groups.

Data	AN	BN	CN	p	η^2^	post-hoc
**N**	25	19	20	-	-	-
**Age [y]**	22±5	22±5	23±3	.715	.01	-
**Education [y]**	14±2	15±2	16±2	.133	.06	-
**BMI [kg/m**^**2**^**]**	16.1±1.0	21.9±2.3	21.5±2.2	**< .001**	.68	AN<BN = CN
Disease duration* **[mos]**	11±6	11±5	-	.985	.01	-
**GM [cc]**	587±42	618±50	601±55	.129	.07	-
**WM [cc]**	497±30	504±44	493±50	.719	.01	-
**CSF [cc]**	212±19	193±22	191±18	.**001**	.20	AN>BN = CN

AN = Anorexia Nervosa, BN = Bulimia Nervosa, CN = Normal controls, values represented mean ± SD.

GM = gray matter, WM = white matter, CSF = cerebrospinal fluid, p = ANOVA probability values for F(2, 61),

* is t(42) and d, in bold FDR q < .05, η^2^ = partial eta square.

All EDI-2 scores were different among groups (see Table 2), except for EDI-2 Maturity Fear. Post-hoc analyses of the significant group effects showed differences in ED compared to CN for all the psychopathological scales with AN, BN>CN (AN = BN). The only exceptions were Body dissatisfaction and Ineffectiveness, with BN>AN>CN and Bulimia with BN>AN, CN (AN = CN). SCL-90 dimensions were all higher in ED compared to CN (see S1 Table).

**Table 2:** Eating disorders inventory 2 scores for the three groups.

Data	AN	BN	CN	p	η^2^	post-hoc
*Drive for Thinness*	14±6	17±6	2±3	**< .001**	.64	AN = BN>CN
*Bulimia*	3±4	12±6	2±2	**< .001**	.53	BN>AN = CN
*Body dissatisfaction*	12±7	21±6	7±6	**< .001**	.46	BN>AN>CN
*Ineffectiveness*	9±5	14±8	4±5	**< .001**	.31	BN>AN>CN
*Perfectionism*	6±4	6±5	3±3	.**040**	.11	AN = BN>CN
*Interpersonal distrust*	7±5	8±4	2±3	**< .001**	.29	AN = BN>CN
*Interoceptive awareness*	9±7	14±8	2±3	**< .001**	.41	AN = BN>CN
*Maturity fears*	8±6	6±5	5±4	.115	.07	-
*Asceticism*	8±5	10±4	3±2	**< .001**	.41	AN = BN>CN
*Impulse regulation*	7±6	8±6	1±1	**< .001**	.32	AN = BN>CN
*Social insecurity*	7±4	9±5	2±3	**< .001**	.35	AN = BN>CN

AN = Anorexia Nervosa, BN = Bulimia Nervosa, CN = Normal controls, values represented mean ± SD.

p = ANOVA probability values for F(2, 58), in bold FDR q < .05, η^2^ = partial eta square.

Self-reported empathy scores did not differ between groups (p = .153). The TAS-20 total score, as well as the Difficulty in identifying and Difficulty in describing feelings subscales, displayed increased alexithymia in AN and BN compared to CN (all p < .001), but no differences were evident between the two clinical groups (all p>.503). See Table 3 for the detailed scores.

**Table 3:** Self-reported empathy, alexithymia, and behavioral performance during affective response task for the three groups.

Data	AN	BN	CN	p	η^2^	post-hoc
**Empathy Quotient**	52±9	48±12	54±9	.153	.06	-
**TAS-20**						
*Difficulty identifying feelings*	23±6	24±6	12±5	**< .001**	.48	AN = BN>CN
*Difficulty describing feelings*	17±5	17±5	11±6	**< .001**	.23	AN = BN>CN
*Externally oriented thinking*	18±4	20±8	15±6	.102	.04	-
*Total score*	59±10	61±15	38±12	**< .001**	.41	AN = BN>CN
**fMRI Affective Responsiveness**						
*Disgust*	9±1	9±2	9±1	.937	.02	-
*Anger*	7±2	7±1	7±1	.249	.04	-
*Fear*	8±1	8±2	8±1	.434	< .01	-
*Total score*	24±3	23±4	24±2	.527	.03	-

AN = Anorexia Nervosa, BN = Bulimia Nervosa, CN = Normal controls, values represent mean ± SD.

TAS-20 = Toronto Alexithymia Scale 20. p = ANOVA probability values for F(2,61), in bold FDR q < .05, η^2^ = partial eta square, for TAS-20 F(2,59), for Empathy Quotient F(2,58).

#### Neuropsychological data

Regarding neuropsychological performance, a significant group effect emerged only for the Stroop Color-Word Test (p < .003). Post-hoc tests indicated that AN and BN showed greater interference than CN (AN>CN, p < .014; BN>CN, p < .004), while the clinical groups did not differ significantly (AN = BN, p < .812). For all other tasks, no significant group difference emerged (all FDR corrected p>.05). Please see S2 Table for details.

#### Behavioral performance during affective responsiveness task

The rmANOVA revealed a significant task effect (p < .001, η^2^ = .46), but no main effect of group (p = .329) and no significant task-by-group interaction (p = .980).

Post-hoc analyses of the Emotion main effect indicated that, when considering the number of correct answers, anger was the most difficult emotion to match (anger<fear, p < .001; anger<disgust, p < .001) and disgust the easiest one (correct matches disgust>anger, p < .001; disgust>fear, p < .008). The control task had a greater number of correct matches than all emotion tasks (all comparison significant, p<0.001). Please see Table 3 for further details and S3 Table for the analysis performed on the errors (group effect not significant).

### Imaging negative emotional situations

#### Task-based fMRI analysis

**Fig 3:**
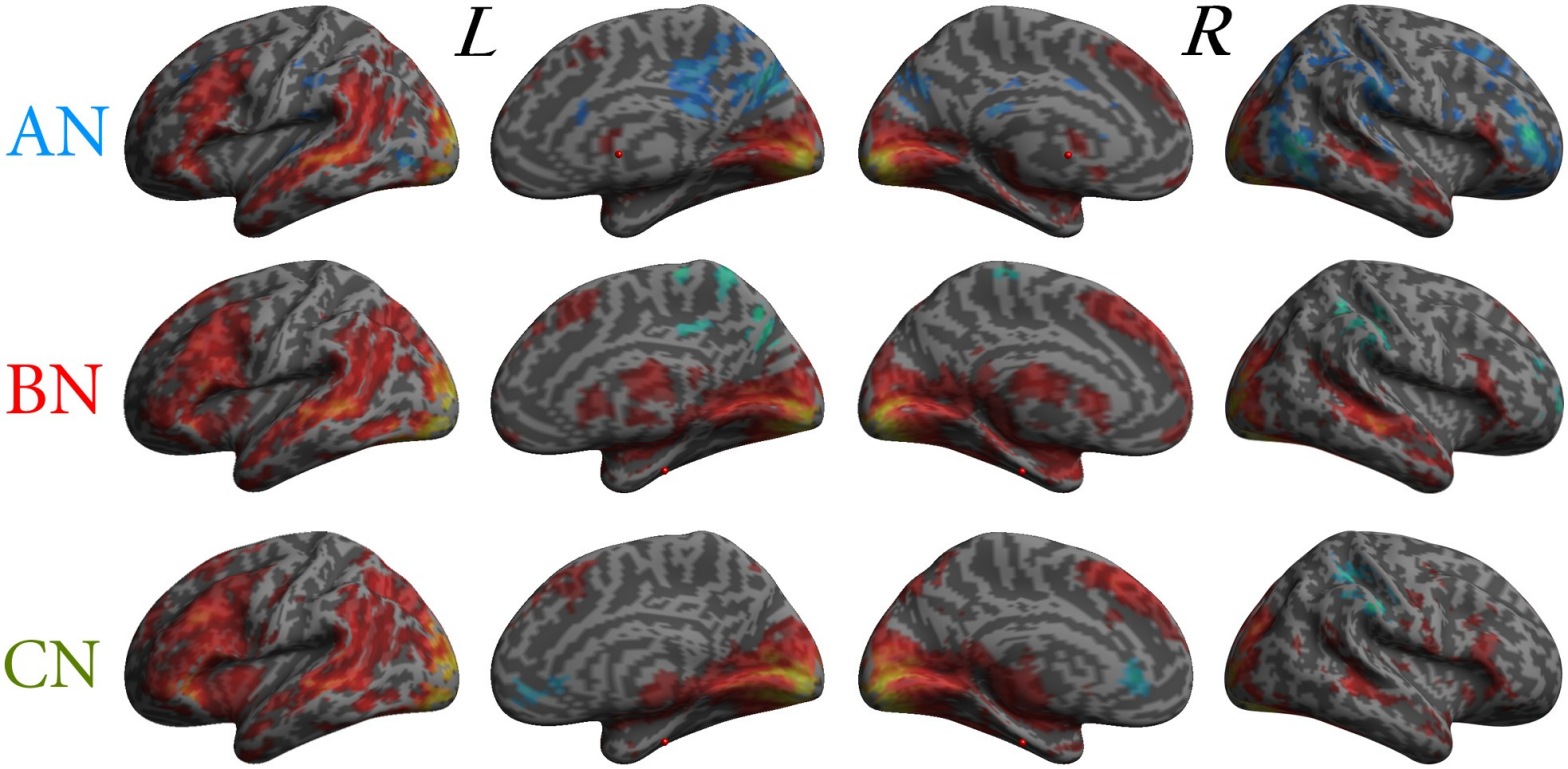
Results of the mean emotional activation and deactivations for the groups. AN = Anorexia Nervosa, BN = Bulimia Nervosa, CN = Normal Controls, L = left, R = right. In yellow-red significant mean activations for t contrast of all groups, in green-blue significant mean deactivations for t contrast of all groups depicted on an inflated 3D brain template (p < .05 FWEc). The shapes of the patterns were qualitatively very similar, but AN showed more extended deactivation, BN wider subcortical activations, CN had the more anterior cluster of deactivation along the midline structures.

The mean effect is shown in Fig 3 for the 3 groups, including both activations (in red) and deactivations (in blue). The patterns were qualitatively similar including a widespread network of activations that included temporal, parietal, frontal, and occipital cortices. We can observe that greater activations were in the visual occipital areas and that the fronto-temporo-parietal activations were mainly in the left hemisphere. The BN group was the only one with an extended activity in the subcortical areas visible in the medial plane.

The deactivations were mainly in the median plane or in the right hemisphere in fronto-parietal areas. Qualitatively the AN group showed more deactivated areas and was the only one presenting some deactivation also in the left hemisphere. ED (AN, and BN) were more deactivated in the posterior part of the midline cortices whereas CN were more deactivated in the anterior portion.

When we compared the differences of activations or deactivations between D, A or F and the neutral condition no significant clusters survived for any group comparison.

#### Multi-voxel pattern analysis

**Fig 4:**
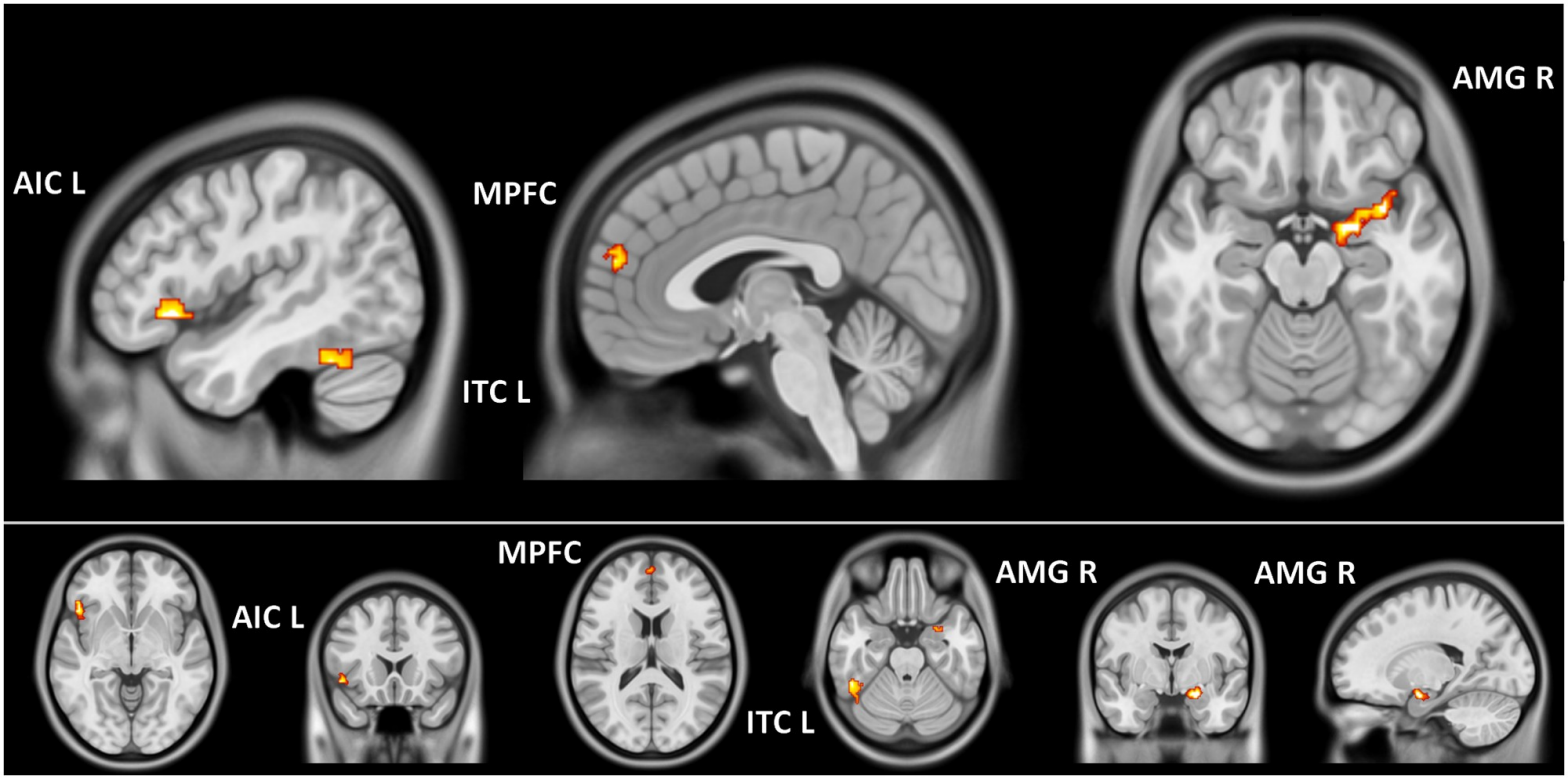
Second-level analysis MVPA. Mean of the three groups for second-level analysis using the SVM weights of all the participants in an ANOVA with three different groups (anorexia, bulimia, controls). Four clusters were important for the different emotions and the neutral conditions: AMG R (right amygdala), AICL (left anterior insula), ITC L (left inferior temporal gyrus), MPFC (middle prefrontal cortex). Clusters depicted on orthogonal projection of an ICBM-152 brain template in neurological convention (p < .05 FWEc).

The most important clusters (Fig 4, Table 4) in the decoding of emotions were:

The right amygdala (this cluster included voxels from the right insula);
The left anterior insula cortex (AIC, this cluster included voxels from the left frontal inferior orbital cortex, BA 47);
The inferior temporal cortex (ITC, this cluster included voxels from the fusiform gyrus);
The frontal superior medial cortex, also called medial prefrontal cortex (MPFC, this cluster included voxel from the anterior cingulum).

**Table 4:** Multi-voxel pattern analyses emotion decoding.

Cluster	Ke	F/t	MNI (mm)			L/R	Region	BA
			x	y	z			
**Mean activation**								
1	156	31.65	28	4	-16	R	Amygdala	-
2	61	30.50	-41	20	-4	L	Insula	-
3	84	28.17	-48	-44	-22	L	Inferior Temporal	20
4	54	27.47	-4	56	18	L	Frontal Superior Medial	10
**BN>CN**								
1	49	9.85	18	-6	-16	R	Amygdala	-
**AN>CN**								
1	33	7.19	0	53	16	L	Anterior Cingulum	32

Ke = cluster extent, x y z = MNI peak coordinates, L = left, R = right, Region = AAL atlas labels, BA = Brodmann Areas. AN = Anorexia Nervosa, BN = Bulimia Nervosa, CN = Normal controls.

Clusters in tables were p < .05 FWEc.

**Fig 5:**
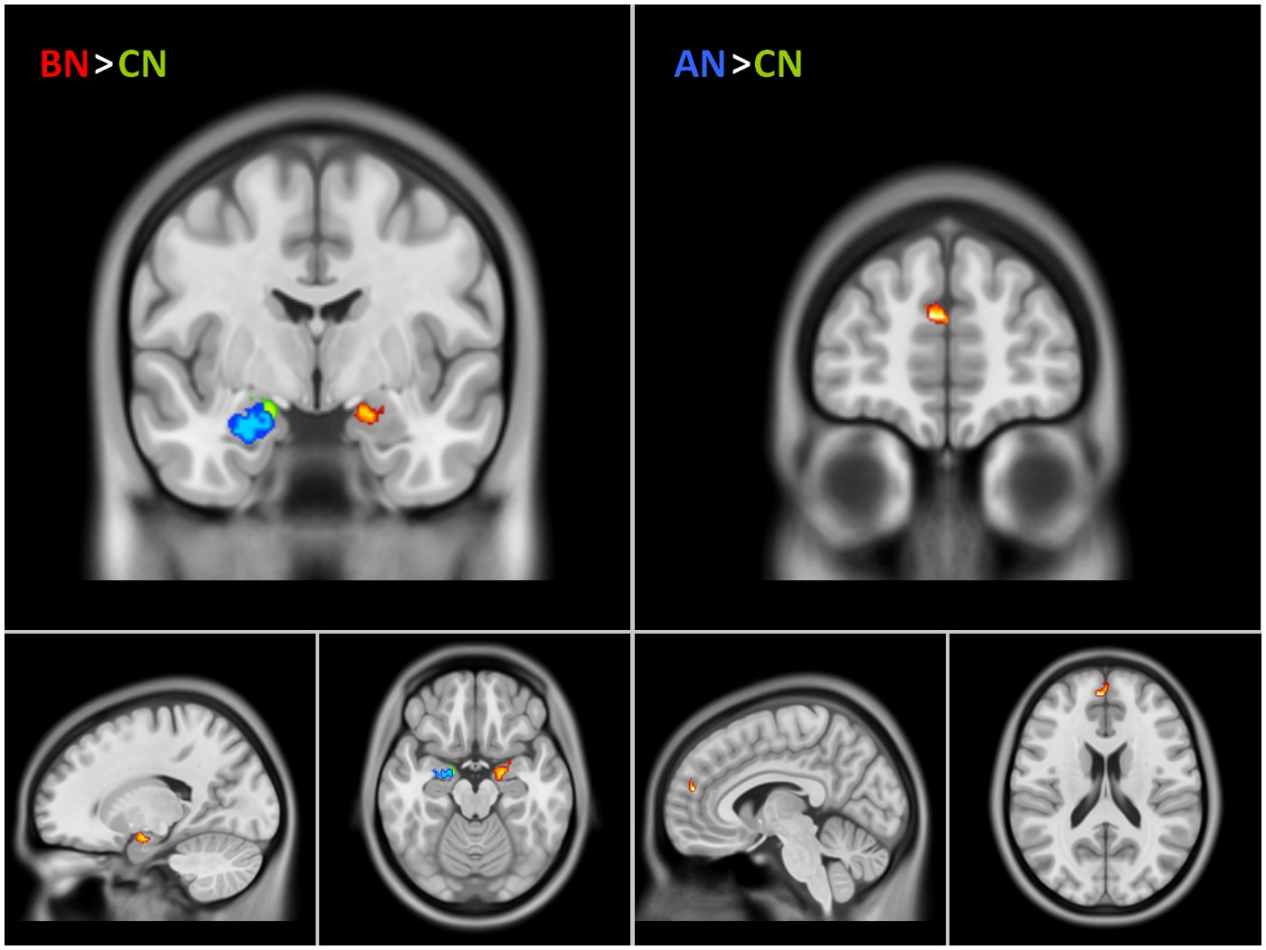
Second-level analysis MVPA group differences. Second-level analysis using the SVM weights, performed as a one-way ANOVA (factor: GROUP. AN = Anorexia Nervosa, BN = Bulimia Nervosa, CN = Normal Controls). Two clusters were more important for decoding the different emotions and the neutral conditions: right centromedial amygdala for BN and MPFC for AN (compared to CN). In light green and blue we show the centromedial and the basolateral portions of the amygdala according to the Pauli atlas [53]. Clusters depicted on orthogonal projection of an ICBM-152 brain template in neurological convention (p < .05 FWEc).

The only pairwise comparison significant were BN>CN and AN>CN (Fig 5, Table 4) in the right amygdala and in the anterior cingulum or anterior cingulate cortex (ACC, BA 32) respectively.

#### Confusion matrix

**Fig 6:**
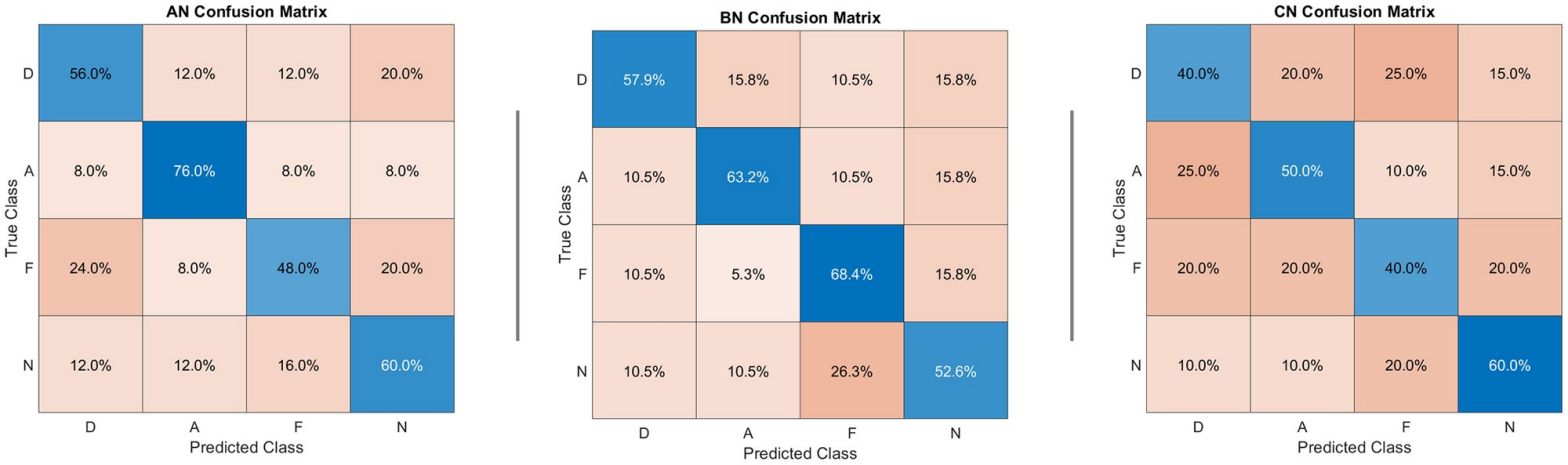
Confusion matrix. AN = Anorexia Nervosa, BN = Bulimia Nervosa, CN = Normal Controls. D = disgust, A = anger, F = fear, N = neutral. Predicted (by the MVPA) vs. real emotion for each stimulus presented during the functional MRI task. No pairwise comparison between matrices is significant.

In Fig 6 confusion matrices for the 3 groups were shown. The homogeneity tests indicated that stimuli labels can be decoded significantly better than chance from the brain activity for AN and BN at a corrected level for every emotion (all p < p_corr_ = .002), but CN had a performance comparable to chance for disgust and fear (Z = 2.76, p = .003 > p_corr_ for both). No pairwise comparison was significant, as the decoding accuracy did not differ between the groups at a p corrected level for all emotions. If we looked for trends, BN had a better performance for fear (Z = 1.86, p = .032 > p_corr_) and AN had a better performance for anger compared to CN (Z = 1.85, p = .032 > p_corr_).

#### Emotion ROI analysis

No ROI pairwise comparison was significant after correction for multiple comparison errors, but we report a trend for BN to have greater activation in the right CM portion of the amygdala only compared with AN for disgust, and only compared with CN for fear (.009 < p_uncorr_ < .003).

#### fMRI relationship with clinical data

**Fig 7:**
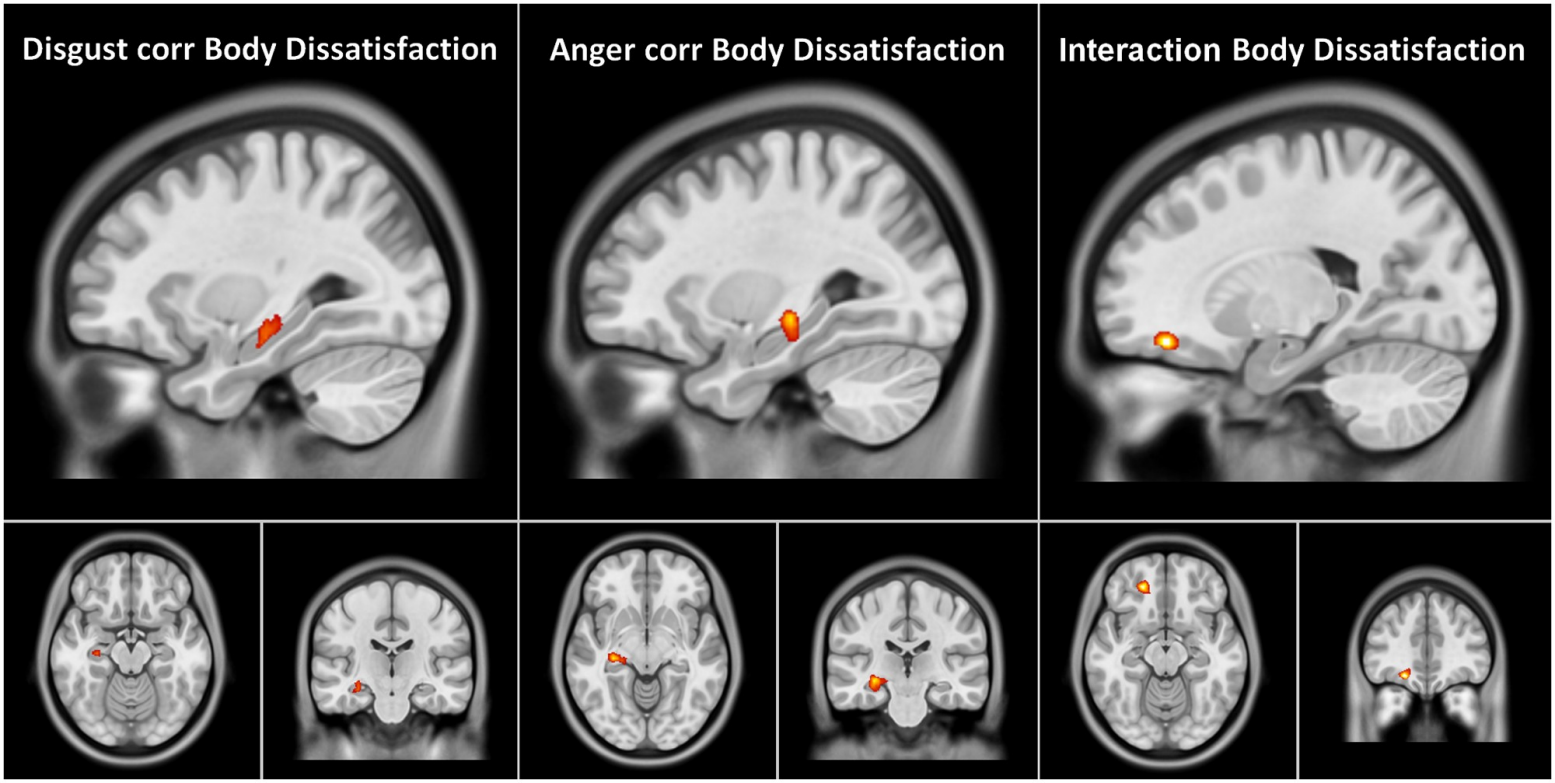
fMRI relationship with clinical data. Correlation between the activation of a cluster in the left hippocampus during disgust and anger stimuli compared to baseline (neutral condition) and the Eating Disorder 2 subscore for Body Dissatisfaction. No interaction emerged except for EDI-2 Body Dissatisfaction score and anger in which a significant interaction was observed in an orbitofrontal cluster only for controls. Clusters depicted on orthogonal projection of an ICBM-152 brain template in neurological convention.

We observed significant negative correlations between the left hippocampus activity during disgust and anger stimuli and the EDI-2 Body Dissatisfaction score (Fig 7, Table 5). No other correlation was found to be significant. Only CN have a significant correlation between the activation signals in the orbitofrontal cluster and EDI-2 Body Dissatisfaction score (Fig 7, Table 5). No other interaction emerged.

**Table 5:** Correlation analysis.

Cluster	Ke	t	MNI (mm)			L/R	Region	BA
			x	y	z			
**Disgust and EDI-2 Body Dissatisfaction**								
1	156	31.65	28	4	-16	L	Hippocampus	-
**Anger and EDI-2 Body Dissatisfaction Interaction**								
1	80	17.55	-20	42	-16	L	Middle Frontal Orbital	11
**Anger and EDI-2 Body Dissatisfaction**								
1	33	7.19	0	53	16	L	Hippocampus	-

Ke = cluster extent, x y z = MNI peak coordinates, L = left, R = right, Region = AAL atlas labels, BA = Brodmann Areas. Clusters in tables were p < .05 FWEc.

## Discussion

This study’s main goal was to address affective responsiveness to negative emotional situations in treatment-naïve young females with AN or BN and matched controls. Thus, this study is one of the few that directly compare neuroimaging activation of different groups of ED with each other, thereby highlighting disorder specific as well as transdiagnostic dysfunctional symptoms.

Based on previous literature we hypothesized to observe significant group differences in empathy and emotion processing between controls and ED. Instead, and in part unexpectedly, we detected mixed results: no significant group differences in behavioural performance or self-reported empathy, but significant differences in alexithymia. We also found no specific differences in neural activation for emotions. As expected, eating-specific and general psychopathology scales sharply distinguished participants affected by ED from healthy controls. Eating-specific scales like Body Dissatisfaction, Ineffectiveness and Bulimia further differentiated the clinical samples, with higher scores in BN.

### Neural network of affective responsiveness in ED

The emotional response paradigm we used involved consistently a set of brain regions [28–30,44], including MPFC, right amygdala and left insula, which have been demonstrated to be involved in emotional empathy [32,36–38] as demonstrated by the MVPA analysis. Interestingly, we did not observe a significant group effect for the amygdala or other brain areas. This lack of a significant group-by-emotion interaction in neural activation during our emotional responsiveness task is partly in line with previous reports of no group differences in neural activation between healthy controls and acute as well as recovered AN for explicit emotion recognition [17–22]. Indeed we observed some relative differences in the importance of the nodes evidenced by MVPA, being ACC more important for AN and right CM amygdala for BN, but the fMRI behavioral recognition task was normal, as the decoding accuracy attested by confusion matrices comparisons. Our results suggest therefore that could be idiosyncrasies in their emotion processing rather than deficits. In fact, characteristic cognitive, temperamental and affective styles have been described for AN and BN [54–56].

The MVPA showed that the decoding accuracy of AN and BN not only was not significantly different from CN, but that there was a trend towards increased accuracy in anger decoding for AN and in fear decoding in BN. Anger has been studied as a sensitive topic for AN [57,58] but fear is not typically associated to BN exception on very specific issues like fear of self, uncertainty or intimacy [59–61].

Qualitatively we also observed some peculiarity in the ED mean deactivations and activations, but it has to be underlined that these observations lie outside the study of emotions or the main aim of this study and disappear when taken into account the neutral condition as reference. The pattern of dectivations in the central midline structures (CMS) is of particular interest, as it belongs to the Default Mode Network, and it was localized more anteriorly in CN. The CMS are important for brain maturation and the reflective functioning and integrity of these processes. Thus, their dysfunction has been associated with problems in the functioning of the self [62]. Dysfunctional activation of these structures has been observed in many psychiatric patients, most frequently in schizophrenia patients reporting lack of frontal cortex deactivation during cognitive and affective tasks [63,64]. Impairments with self-reference or relational aspects of the self may contribute to the neural alteration, though no behavioral effect emerged. Problems with self-reference are in line with previous results, where alterations in emotion recognition accuracy in AN were only apparent when the image depicted was the own face [18].

Commenting on the trends evidenced, BN showed some relevant activation in the CM portion of the right amygdala, both CM and BL respond to aversive stimuli, but CM has been reported as the primary output structure, important for the arousal and fear response activation [33,65]. Differences in the amygdala functioning in the context of ED are important since this structure has a role in reward mechanisms and opioid-mediated food pleasure [66,67].

We found a negative correlation between the activity of the left hippocampus and Body Dissatisfaction. We postulate that this result can be explained by the reduced hippocampal activity when subjects are faced with unpleasant images or in the presence of negative emotions, as shown by fMRI studies [68,69], and that Body Dissatisfaction, a negative emotion and a core pathological trait for ED could increase the magnitude of this effect. As this result was found to hold only for the left hippocampus, however, the hypothesis needs further corroboration. The additional correlation found between anger and Body Dissatisfaction in the orbitofrontal cortex is not surprising as the hippocampus is functionally connected with this area during anger feeling [70], but it is noteworthy that was observable only in CN and not in ED. We can speculate that the normal network is less recruited in ED with high Body Dissatisfaction scores, and so less detectable, but the hypothesis should be investigated with more focus in future.

### Empathy and affective responsiveness in ED

Previous findings on empathy or emotion recognition are inconsistent: while some reported decreased self-reported emotional empathy but normal emotion recognition in acute AN [12], others observed no significant alteration in self-reported empathy in AN [13]. Additionally, a significant impairment in understanding others’ emotions and in the regulation of their own emotions has been reported in AN [57,71]. However, studies based on film clips did not find any specific abnormalities in emotion processing and emotional ratings, only attentional biases [72]. Some researchers pointed out that at least in a subgroup of individuals affected by BN, empathic mentalization was not impaired at all [73,74].

It has been hypothesized that the mixed results derived from the relatively poor sensitivity and specificity of the applied instruments, as already evidenced in the literature [42], but also considering our negative findings with a highly sensitive measurement of the brain activity it seems an incomplete explanation. A recent review [14] suggests an alternative explanation: ED individuals can recognize others’ basic emotions, but they lose this skill when emotions become more complex and are expressed within a relationship. Relevant to this kind of hypothesis, a dysfunctional middle prefrontal cortex (MPFC) activation has been reported for the processing of visual stimuli depicting couples in intimate relationships in acute and recovered AN, pointing to a state-independent alteration of MPFC activation [75,76]. Additionally, the significantly elevated alexithymia scores in both clinical groups may be also associated with MPFC dysfunction, as this is an important hub for the interhemispheric integration and transfer [77]. Thus, MPFC function may be compromised in ED indicating a transdiagnostic alteration that seems especially relevant for the processing of complex stimuli, social functioning, and interpersonal interactions.

### Limitations

Despite the novelty and relevance of this investigation of emotional responsiveness, the study had several limitations that need to be acknowledged. First, we only investigated a small fragment of emotion processing and social cognition. More distinct differences between ED and controls could emerge if other processes that vary in complexity would be studied like affective vs. cognitive empathy or interpersonal affectivity and reactivity. Second, different subtypes of participants (purging, non-purging) were included, but subsamples were too small to analyse their specific contributions. Finally, the limited size of our sample limits the strength of the conclusions that can be drawn from our results. It also caused the impossibility of studying the specific contributions of different subtypes of anorexia nervosa (binging-purging, restricting…). This study could therefore be considered a first step, and investigations enrolling a larger number of patients are therefore warranted.

## Conclusions

Using both self-report data and an affective responsiveness task, we did not find data in support of the hypothesis that participants with ED display a reduced ability to recognize emotions. We however recognize that the limited power due to our sample size might be responsible for some of our null results, especially the ones regarding our fMRI task. Furthermore, while we acknowledge that other studies did find differences in brain activity during the emotional appraisal of different stimuli, like food and non-food pictures, the same authors failed to find the same results in a replication attempt with a larger group [78], therefore proving that further and more powerful investigations are needed before a sharp conclusion on this matter can be drawn.

As we did not find evidence in support of an impaired emotion processing, we propose further studies that directly investigate alternative theories (e.g., the role of attachment processes in the pathogenesis of anorexia nervosa [79–81]), rather than from deficits in emotion processing and emotional responsiveness.

If first episode, treatment-naive AN and BN patients do not suffer from a difficulty in feeling and identifying basic negative emotions, it is unlikely that relational obstacles, including the ones encountered in a psychotherapeutic journey, are secondary to impaired recognition of emotions. In this light, our preliminary findings suggest that focusing the therapy on learning basic emotion processing could be an inadequate strategy.

## Supporting information

S1 Table Symptom checklist-90 data for the three groups. (DOCX)

S2 Table Neuropsychological data for the three groups. (DOCX)

S3 Table fMRI affective responsiveness errors. (DOCX)

S1 File Supplementary materials and methods. (DOCX)
