# Supplementary material for: Emotional imagination of negative situations: Functional neuroimaging in anorexia and bulimia
Source: PLoS One. 2021 Apr 9;16(4):e0231684. doi: 10.1371/journal.pone.0231684 (PMC8034744; doi:10.1371/journal.pone.0231684)
Supplement: S1 Table — (DOCX) [file pone.0231684.s001.docx]

**Table S1. Symptom Checklist-90 data for the three groups**

| **Data** | **AN** | **BN** | **CN** | **p** | **η^2^** | **post-hoc** |
| --- | --- | --- | --- | --- | --- | --- |
| *Somatization* | 20±8 | 21±8 | 10±5 | **<.001** | .31 | AN=BN>CN |
| *Obsessive-compulsive* | 16±8 | 19±8 | 8±5 | **<.001** | .32 | AN=BN>CN |
| *Interpersonal sensitivity* | 15±7 | 19±7 | 6±5 | **<.001** | .41 | AN=BN>CN |
| *Depression* | 24±10 | 29±10 | 9±6 | **<.001** | .47 | AN=BN>CN |
| *Anxiety* | 18±7 | 21±8 | 7±5 | **<.001** | .43 | AN=BN>CN |
| *Hostility* | 10±5 | 11±6 | 2±2 | **<.001** | .37 | AN=BN>CN |
| *Phobic Anxiety* | 5±3 | 8±5 | 2±2 | **<.001** | .34 | AN=BN>CN |
| *Paranoid ideation* | 8±5 | 10±6 | 4±3 | **<.001** | .27 | AN=BN>CN |
| *Psychoticism* | 10±7 | 13±6 | 2±2 | **<.001** | .41 | AN=BN>CN |

AN = Anorexia Nervosa, BN = Bulimia Nervosa, CN = Normal controls, values represented mean ± SD, p = ANOVA probability values for F(2, 58), in bold FDR q<.05, η^2^ = partial eta square
