# Supplementary material for: Emotional imagination of negative situations: Functional neuroimaging in anorexia and bulimia
Source: PLoS One. 2021 Apr 9;16(4):e0231684. doi: 10.1371/journal.pone.0231684 (PMC8034744; doi:10.1371/journal.pone.0231684)
Supplement: S2 Table — (DOCX) [file pone.0231684.s002.docx]

**Table S2. Neuropsychological data for the three groups**

| Test | AN | BN | CN | p | η^2^ | post-hoc |
| --- | --- | --- | --- | --- | --- | --- |
| Digit Span | 6.7±1.0 | 6.2±0.8 | 7.2±2.5 | .145 | .06 | - |
| Digit Back | 5.3±1.0 | 4.7±1.0 | 4.8±1.1 | .140 | .07 | - |
| Corsi Span | 5.5±0.9 | 5.4±0.9 | 5.4±1.2 | .973 | <.01 | - |
| Stroop CW | 102±20 | 107±17 | 81±35 | **.003** | .17 | AN=BN>CN |
| TMT-A | 33±12 | 31±9 | 30±7 | .567 | .02 | - |
| TMT-B | 54±19 | 58±16 | 51±9 | .423 | .03 | - |
| RAVLT IR | 62±7 | 55±9 | 62±8 | .019 | .12 | - |
| RAVLT DR | 14±2 | 12±2 | 13±1 | .015 | .13 | - |
| Fluency Pho | 48±11 | 44±12 | 43±11 | .264 | .04 | - |
| WAIS Voc | 53±10 | 53±15 | 53±11 | .973 | <.01 | - |
| WCST Cat | 6.0±0.2 | 5.3±1.8 | 6.0±0.0 | .058 | .09 | - |
| WCST Pers | 7±6 | 13±13 | 7±3 | .050 | .10 | - |
| WCST Err | 5±4 | 10±18 | 6±3 | .209 | .05 | - |
| WAIS Block | 40±8 | 38±7 | 38±7 | .403 | .03 | - |
| fMRI Info Proc | 9±1 | 9±1 | 10±0 | .243 | .04 | - |

AN = Anorexia Nervosa, BN = Bulimia Nervosa, CN = Normal controls, N.S. = not significant CW = colored word, TMT = Trial Making Test, RAVLT = Rey Auditory Verbal Learning Test, IR = immediate recall, DR = Delayed Recall, Pho = phonemic, WAIS = Wechsler Adult Intelligence Scale, Voc = vocabulary, WCST = Wisconsin Card Sorting Test, Cat = categories, Pers = perseverations, Err = errors, Info Proc = Information processing; p = ANOVA probability values for F(2, 61), in bold FDR q<.05, η^2^ = partial eta square, F(2,59) for Digit, F(2,60) for Corsi Span, F(2,59) for WCST
