# Supplementary material for: Emotional imagination of negative situations: Functional neuroimaging in anorexia and bulimia
Source: PLoS One. 2021 Apr 9;16(4):e0231684. doi: 10.1371/journal.pone.0231684 (PMC8034744; doi:10.1371/journal.pone.0231684)
Supplement: S3 Table — (DOCX) [file pone.0231684.s003.docx]

**Table S3. fMRI affective responsiveness errors**

| **Data** | **AN** | **BN** | **CN** | **p** | **η^2^** | **post-hoc** |
| --- | --- | --- | --- | --- | --- | --- |
| *Disgust errors* | 1±1 | 2±2 | 1±1 | .507 | .02 | - |
| *Anger errors* | 3±2 | 3±1 | 3±1 | .520 | .02 | - |
| *Fear errors* | 2±1 | 2±2 | 2±1 | .905 | <.01 | - |
| *Total errors* | 6±3 | 7±4 | 6±2 | .482 | .02 | - |

AN = Anorexia Nervosa, BN = Bulimia Nervosa, CN = Normal controls, values represented mean ± SD, p = ANOVA probability values for F(2, 61), η^2^ = partial eta square
