# Supplementary material for: Emotional imagination of negative situations: Functional neuroimaging in anorexia and bulimia
Source: PLoS One. 2021 Apr 9;16(4):e0231684. doi: 10.1371/journal.pone.0231684 (PMC8034744; doi:10.1371/journal.pone.0231684)
Supplement: S1 File — (DOCX) [file pone.0231684.s004.docx]

# S1 Supplementary Materials and Methods

## S1.1 Clinical, neuropsychological and psychological assessment

### S1.1.1 Self-reported clinical scales

Patients and controls filled out the clinical scales described below:

The *Eating Disorder Inventory 2* (EDI-2) is a self-report questionnaire with 91 items designed to assess the presence of ED. It has good reliability and validity (1,2) and assesses 11 dimensions: Drive for thinness, Bulimia, Body dissatisfaction, Ineffectiveness, Perfectionism, Interpersonal distrust, Interoceptive awareness, Maturity fears, Asceticism, Impulse Regulation, and Social Insecurity.

The *Symptom Checklist-90* (SCL-90) is a self-report questionnaire with 90 items designed to evaluate a broad range of psychological problems and psychopathological symptoms. It has good to excellent reliability and validity (3,4) and assesses 9 dimensions: somatization, obsessive-compulsive, interpersonal sensitivity, depression, anxiety, hostility, phobic anxiety, paranoid ideation, psychoticism.

### S1.1.2 Neuropsychological tests

A variety of cognitive abilities were assessed in our sample using standardized Italian versions: working memory (Digit Span Backward (5)), verbal and spatial attention spans (Digit Span and Corsi block tapping test (6,7)), information processing (TMT-A (8)), interference resistance (Stroop Test (9)), short and long term verbal memory (RAVLT (10,11)), language functions (Vocabulary WAIS (5) and Phonemic Fluency (12)), visuospatial constructive skills (Block Design WAIS (5)) and executive functioning (TMT-B (8), WCST (13)). Data were compared with results from normative Italian samples (10,14,15), using correction grids for age and education, and identifying the individuals with performances under the cutoffs of normality and at a level of clinical interest.

### S1.1.3 Self-reported empathy and alexithymia

Patients and controls filled out the following self-report questionnaires:

The *Empathy Quotient* (EQ) has 60 items designed to measure temperamental empathy in adults with good validity and reliability. It can detect temperamental differences between genders and defines empathy as including a cognitive, an affective and a social component (16,17).

The *Toronto Alexithymia Scale* (TAS-20) is commonly used to investigate alexithymia, characterized by internal consistency, high test–retest reliability, construct and criterion validity, both in normal and clinical samples (18,19). Alexithymia is a deficit in identifying, describing and reporting own and other people’s feelings. TAS-20 assesses 3 dimensions: Difficulty Describing Feelings (DDF), Difficulty Identifying Feelings (DIF) and Externally-Oriented Thinking (EOT) (20–22).

## S1.2 Experimental design

First the enrolled patients had a clinical assessment where the diagnosis was confirmed and clinical parameters collected. After one week, time useful to check all the exclusion criteria, the neuropsychological data were assessed and the fMRI session was performed.

Besides the SCID, the clinical assessment included a complete anamnesis, EDI-2 and SCL-90 scales administration, and the neuropsychological test battery was completed (as described above). The battery was administered in the morning (between 10 am and 11.30 am) and lasted about 90 minutes.

Patients underwent fMRI scanning in the afternoon (between 17.30 pm and 18.00 pm), for a total time of about half an hour. The functional paradigm was followed by a high resolution anatomical T1-weighted sequence. Between the two session (clinical/neuropsychological assessment and MRI session), participants generally went to their houses and come back to finish the experiment.

Participants were instructed to report anxiety feelings during scanning on a scale ranging from no anxiety, to minimal tolerable anxiety, mildly disturbing, disturbing, severe anxiety, panic attack. They were asked to do that at the end of the protocol. Five participants reported minimal tolerable anxiety (2 BN, 3 CN); all others reported no anxiety.

# Supplementary References

1. Garner DM, Olmstead MP, Polivy J. Development and validation of a multidimensional eating disorder inventory for anorexia nervosa and bulimia. International Journal of Eating Disorders. 1983;2(2):15–34.

2. Garner DM. Eating Disorder Inventory 2: professional manual. Odessa: Psychological Assessment Resources; 1991.

3. Derogatis LR, Rickels K, Rock AF. The SCL-90 and the MMPI: a step in the validation of a new self-report scale. The British journal of psychiatry: the journal of mental science. 1976;128:280–9.

4. Derogatis LR, Lipman RS, Covi L. SCL-90: an outpatient psychiatric rating scale--preliminary report. Psychopharmacology bulletin. 1973;9(1):13–28.

5. Wechsler D. The Wechsler Adult Intelligence Scale: Italian edition. Firenze: Organizzazioni Speciali; 1974.

6. Miller GA. The magical number seven plus or minus two: some limits on our capacity for processing information. Psychological review. 1956;63(2):81–97.

7. Orsini A, Grossi D, Capitani E, Laiacona M, Papagno C, Vallar G. Verbal and spatial immediate memory span: normative data from 1355 adults and 1112 children. Italian journal of neurological sciences. 1987;8(6):539–48.

8. Giovagnoli AR, Del Pesce M, Mascheroni S, Simoncelli M, Laiacona M, Capitani E. Trail making test: normative values from 287 normal adult controls. Italian journal of neurological sciences. 1996;17(4):305–9.

9. Brugnolo A, De Carli F, Accardo J, Amore M, Bosia LE, Bruzzaniti C, et al. An updated Italian normative dataset for the Stroop color word test (SCWT). Neurological sciences: official journal of the Italian Neurological Society and of the Italian Society of Clinical Neurophysiology. 2015; 37(3):365–72.

10. Lezak MD. Valutazione neuropsicologica. 4th ed. /. Milano: Edra; 2004. xiv, 1016 p.

11. Ray RD, Zald DH. Anatomical insights into the interaction of emotion and cognition in the prefrontal cortex. Neuroscience and biobehavioral reviews. 2012;36(1):479–501.

12. Novelli G, Papagno C, Capitani E, Laiacona N, Vallar G, Cappa SF. Tre test clinici di ricerca e produzione lessicale. Taratura su sogetti normali / Three clinical tests to research and rate the lexical performance of normal subjects. Archivio di Psicologia, Neurologia e Psichiatria. 1986;47(4):477–506.

13. Laiacona M, Inzaghi MG, De Tanti A, Capitani E. Wisconsin card sorting test: a new global score, with Italian norms, and its relationship with the Weigl sorting test. Neurological sciences: official journal of the Italian Neurological Society and of the Italian Society of Clinical Neurophysiology. 2000;21(5):279–91.

14. Capitani E, Laiacona M. Composite neuropsychological batteries and demographic correction: standardization based on equivalent scores, with a review of published data. The Italian Group for the Neuropsychological Study of Ageing. Journal of clinical and experimental neuropsychology. 1997;19(6):795–809.

15. Spinnler H, Tognoni G. Standardizzazione e taratura italiana di test neuropsicologici. Ital J Neurol Sci. 1987;6 (suppl 8).

16. Lawrence EJ, Shaw P, Baker D, Baron-Cohen S, David AS. Measuring empathy: reliability and validity of the Empathy Quotient. Psychological medicine. 2004;34(5):911–9.

17. Baron-Cohen S, Wheelwright S. The empathy quotient: an investigation of adults with Asperger syndrome or high functioning autism, and normal sex differences. Journal of autism and developmental disorders. 2004;34(2):163–75.

18. Bagby RM, Parker JD, Taylor GJ. The twenty-item Toronto Alexithymia Scale--I. Item selection and cross-validation of the factor structure. Journal of psychosomatic research. 1994;38(1):23–32.

19. Bagby RM, Taylor GJ, Parker JD. The Twenty-item Toronto Alexithymia Scale--II. Convergent, discriminant, and concurrent validity. Journal of psychosomatic research. 1994;38(1):33–40.

20. Bressi C, Taylor G, Parker J, Bressi S, Brambilla V, Aguglia E, et al. Cross validation of the factor structure of the 20-item Toronto Alexithymia Scale: An Italian multicenter study. Journal of Psychosomatic Research. 1996;41:551–9.

21. Taylor GJ, Bagby RM, Parker JDA. The 20-Item Toronto Alexithymia Scale: IV. Reliability and factorial validity in different languages and cultures. Vol. 55, Journal of Psychosomatic Research. 2003. p. 277–83.

22. Parker JDA, Taylor GJ, Bagby RM. The 20-Item Toronto Alexithymia Scale: III. Reliability and factorial validity in a community population. Journal of Psychosomatic Research. 2003;55:269–75.
